# Low Fidelity Trainer for Fiberoptic Scope Use in the Emergency Department

**DOI:** 10.21980/J8764B

**Published:** 2020-07-15

**Authors:** Garren Giles, Dominic Diprinzio, Jordana Haber

**Affiliations:** * University of Nevada, Las Vegas School of Medicine, Department of Emergency Medicine, Las Vegas, NV

## Abstract

**Audience:**

The low fidelity trainer for fiberoptic scope is designed to train emergency medicine (EM) residents PGY I-IV, and medical students interested in EM.

**Introduction:**

Fiberoptic intubation is a skill that Emergency Medicine (EM) providers should be familiar with, though it is a rarely encountered procedure in the clinical setting. Approximately 1% of endotracheal intubations are performed using fiberoptic intubation.^1^,^2^ The success rate of first attempt fiberoptic intubation is about 50%. When fiberoptic intubation is used as a rescue device it has been shown to be about 70 % successful.^1^ Despite being an important skill for emergency physicians to have, fiberoptic intubation competency is not required during emergency medicine residency^1^ and resident physicians have limited exposure to learning this life-saving skill.

Given that fiberoptic intubation is a rarely performed procedure in the clinical setting, the most practical way for EM learners to gain proficiency would be through simulation. The use of fiberoptic trainers in anesthesiologist resident training has shown improvement in first pass success and overall skills with using the fiberoptic scope.^3^–^6^ Simulation has also been shown to improve resident procedural knowledge and skills in many other fields.^7^–^11^ Simulation offers training with seldom performed procedures, and there is evidence that simulation does improve patient outcomes and reduce errors.^2^,^12^–^14^ In order to help EM learners gain confidence and increase their comfort in using the fiberoptic scope, we developed a low fidelity training model that allows the learner to practice fiberoptic intubation.

**Educational Objectives:**

By the end of this training session, learners will be able to 1) list indications, contraindication, and complications in performing fiberoptic intubations, 2) know how to use and maneuver a fiberoptic scope, and 3) be able to successfully intubate the trainer model.

**Educational Methods:**

The training model consists of a large 55-gallon tote with polyvinyl chloride (PVC) pipes enclosed in the tote. The pipes were arranged in various manners: several pipes simulated the oropharynx and trachea, and others were arranged into a series of mazes, to require the learner to manipulate the scope through the maze to reach the end. The multiple stations within the model provided ample opportunity for the learner to acquire confidence with the fiberoptic scope and the movements required to maneuver the scope into position.

**Research Methods:**

The model was used in our weekly Emergency Medicine conference during a low fidelity simulation day. The residents were split into groups consisting of 5–6 learners. The residents and medical students were given a brief 5-minute lecture on fiberoptic intubation, which reviewed indications for fiberoptic intubation, and a demonstration on how to operate the fiberoptic scope. Following the briefing, each group had approximately 25–30 minutes to practice using the simulated fiberoptic scope model. Each learner in the group was then encouraged to practice navigating the other mazes at their own discretion. Residents and medical students were given a survey before and after using the fiberoptic training model to assess their knowledge and confidence in performing the procedure.

**Results:**

The use of the fiberoptic trainer was successful in helping learners to become more familiar with the fiberoptic scope and learn the skills to maneuver the scope successfully through the trainer. Each resident performed a survey prior to and after the fiberoptic instruction and training. They were asked to rate their confidence in identifying airway landmarks, perform the procedure without supervision, and identify correct supplies needed for procedure. All areas increased in confidence except in identifying correct supplies for PGY-II (−0.1), and PGY-III (−0.4). The greatest increase was amongst PGY-I residents in confidence identifying airway landmarks, with an increase of 4.2.

**Discussion:**

As with any simulation model, this model does not perfectly recreate human anatomy. For example, in our model there were no simulated secretions or blood. The actual appearance of the anatomy will be very different from that which was used in our model, which may lead to an unsuccessful intubation attempt. The scenarios in which we were using the trainer was a low stress environment, unlike the usual emergency setting. Despite this, there was an increase in learner confidence in using a fiberoptic scope to manage emergency airways. It also offered a unique experience and gave the learners an opportunity to learn how to manipulate the fiberoptic scope that a traditional high fidelity model may not offer. Future comparisons could be made between a low fidelity simulation and high fidelity simulation device, and the addition of simulated secretions could help increase learning and confidence in fiberoptic intubations.

**Topics:**

Difficult airway management, Fiberoptic intubation, fiberoptic use in emergency department.

## USER GUIDE

[Table t1-jetem-5-3-i1]

**Table t1-jetem-5-3-i1:** 

List of Resources:	
Abstract	1
User Guide	3
Instructor Guide	8

**Learner Audience:**

Medical Students, Interns, Junior Residents, Senior Residents

**Time Required for Implementation:**

Time to build trainer takes approximately 2 hours. Learning session should take 45 minutes. 15-minute introduction to fiberoptic scope and trainer. 25 minutes hands-on use of fiberoptic scope with trainer. 5-minute final review

**Recommended Number of Learners per Instructor:**

Learner to instructor ratio will depend on how many fiberoptic scopes are available. It is recommended to not exceed a 4:1 ratio in order to allow adequate time spent with each learner. The trainer has multiple practice ports that may be used individually by each learner.

**Topics:**

Difficult airway management, Fiberoptic intubation, fiberoptic use in emergency department.

**Objectives:**

By the end of the instruction learners should:

1. Know the indications, contraindications, and complications for fiberoptic intubation in the emergency department
2. Understand how to use a fiberoptic scope
3. Be able to maneuver the fiberoptic scope more easily
4. Be able to successfully intubate trainer with ETT (endotracheal tube)

### Linked objectives and methods

Instructor will go over all indications and complications with fiberoptic intubations in a discussion format, allowing for questions (Objective 1). The Instructor will then go over how to use a fiberoptic scope properly; this will be performed by demonstrating how to hold a fiberoptic scope, different ways to maneuver the scope, how to visualize what is being projected on the screen, and how the scope will be used for intubations (Objective 2). Each learner will then take a practice scope and use it without the trainer, in order to get the correct feel for each scope and see how to maneuver the tip of the scope with the controller. Learners will then apply this in the trainer and maneuver around the PVC pipe maze and obstacles placed in the maze while the instructor individually helps each learner and answers questions that may arise while using the trainer (Objective 3). Finally, each learner will use one of the two simulated airways on the top of the tote to advance an ETT in the trachea (Objective 4).

### Recommended pre-reading for instructor

The instructor should find the fiberoptic scope used in their department and use the scope. Know and understand how the scope works and become familiar with how to use it in order to teach it to the learners.

- Read: Driver, BE, Reardon RF, Flexible Endoscopic Intubation. In: Robert JR, Custalow CB, Thomsen TW, Hedghes JR, eds. *Roberts and Hedges’ Clinical Procedures in Emergency Medicine and Acute Car*e. 6th ed. Philadelphia, PA: Elsevier; 2013: 95–101.
- Read: Hayden EM, Pallin DJ, Wilcox SR, et al. Emergency department adult fiberoptic intubations: incidence, indications, and implications for Training. *Acad Emerg Med*. 2018;25(11):1263–1267. doi:10.1111/acem.13440. Accessed 6/30/2020.
- Read: Simma L, Cincotta D, Sabato S, Long E. Airway emergencies presenting to the paediatric emergency department requiring advanced management techniques. *Arch Dis Child*. 2017;102(9):809–812. doi:10.1136/archdischild-2016-311945.

### Learner responsible content (LRC)

- Driver, BE, Reardon RF, Flexible Endoscopic Intubation. In: Robert JR, Custalow CB, Thomsen TW, Hedghes JR, eds. *Roberts and Hedges’ Clinical Procedures in Emergency Medicine and Acute Car*e. 6th ed. Philadelphia, PA: Elsevier; 2013: 95–101.
- Hayden EM, Pallin DJ, Wilcox SR, et al. Emergency department adult fiberoptic intubations: incidence, indications, and implications for Training. *Acad Emerg Med*. 2018;25(11):1263–1267. doi:10.1111/acem.13440.
- Simma L, Cincotta D, Sabato S, Long E. Airway emergencies presenting to the paediatric emergency department requiring advanced management techniques. *Arch Dis Child*. 2017;102(9):809–812. doi:10.1136/archdischild-2016-311945.

### Implementation Methods

- Trainer is best in a small group setting no more than 4:1 learner to instructor ratio
- Small groups will discuss the indications, contraindications, and complications to performing a fiberoptic intubation.
- Instructors will gauge learners’ understanding and comfort in using a fiberoptic scope through group discussion.
- Instructor will then demonstrate how to use a fiberoptic scope and how to intubate using a fiberoptic scope on trainer.
- Learners will have hands-on experience with practice fiberoptic scopes though the PVC mazes while instructor answers questions and provides real time feedback.
- Learners will then pass an ETT through simulated airway on trainer while instructor provides real time feedback.
- Through small group discussions any additional questions will be answered, and instructor will review key points of training session.

### List of items required to replicate this innovation

All items may be purchased at any local home improvement store:

1. Tote ($25.00)
2. 2: ½”x10’ PVC pipe ($4.40)
3. 5: ½” PVC running trap ($9.00)
4. 5: ½” EL 90 degree with outlet PVC connector ($4.50)
5. 2: ¾” coupling PVC connector ($0.64)
6. 13: ½” EL 45 degree PVC connectors ($11.05)
7. 4: ½” Cross PVC connector ($5.72)
8. 2: ¾” IL 45 degree PVC connector ($2.28)
9. 6: ½” PVC caps ($2.70)
10. 3: ½” TEE PVC connector ($1.62)
11. 2: ¾”x ½” PVC connector ($1.18)
12. 1: ¾”x 2’ PVC Pipe ($1.18)
13. 2: ½” PVC Cap ($0.90)
14. 3: ½” PVC trap ($5.40)
15. 6: ½” EL 45 degree PVC connector ($5.10)
16. PVC glue ($5.00)
17. Hot glue gun ($9.00, Amazon.com)
18. Hot glue sticks ($6.00)
19. Standard handheld electrical drill (Price varies)
20. 1” Spade drill bit
21. ¾” Spade drill bit
22. Hack saw (Price Varies)
23. 6.0–8.0 ETT (Hospital acquired)
24. Fiberoptic scope (Varies depending on what your ED has available)

Cost will vary depending on size of tote, number of PVC pipes, and connectors you desire to have in your fiberoptic trainer maze.

### Approximate cost of items to create this innovation

Total cost for this particular trainer was $110.00; this can vary based on type of tote purchased and how many different PVC pipes and connectors are used. The cost of the fiberoptic scope is not included in this price. This will be the largest cost for the simulation. It is recommended that the fiberoptic scope be acquired by your hospital in order for EM learners to learn on the fiberoptic scope they will use in their emergency department. In addition, there are opportunities to speak with fiberoptic scope representatives in order to purchase practice scopes. Once items are purchased, the PVC pipes are able to be re-arranged in multiple configurations in order to practice different types of maneuvers with the fiberoptic scope.

### Detailed methods to construct this innovation

#### Mazes

1. Mark out multiple side ports.
2. Using the ¾” spade drill bit to drill the desired side holes in the tote.[Fig f1-jetem-5-3-i1]
  **Figure f1-jetem-5-3-i1:**
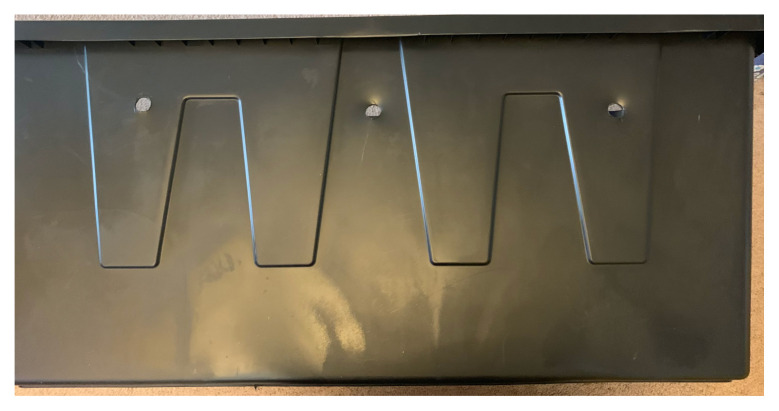
3. Using the hack saw cut each PVC pipe in various lengths not to exceed the width or length of the tote.[Fig f2-jetem-5-3-i1]
  **Figure f2-jetem-5-3-i1:**
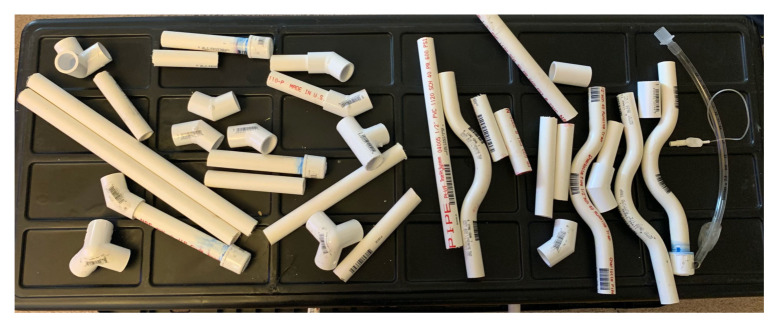
4. Place ½” PVC pipes in side holes and use them as the beginning ports of each maze[Fig f3-jetem-5-3-i1]
  **Figure f3-jetem-5-3-i1:**
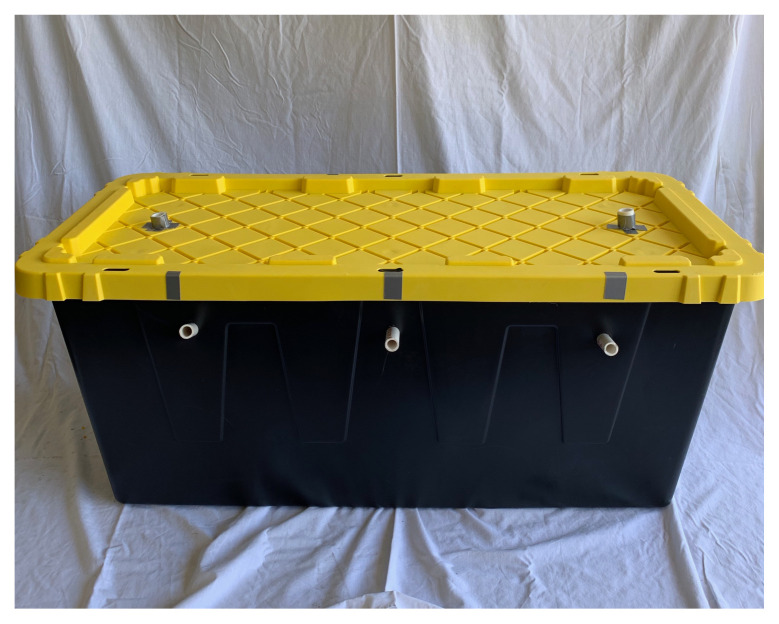
5. In different PVC cut outs, use hot glue to add obstacles, allow them to dry completely.
6. Take 4–5 ½” PVC pipes and glue on ½” caps; these will be the end of one of the portions of the maze. You may add a picture, shape, or color to the cap to make the end of the maze more interesting.
7. Using PVC traps, connectors, and various length pipes, connect them in order to make different mazes from each port. Do not glue pipes or connectors together in order to allow different configurations of mazes.[Fig f4-jetem-5-3-i1]
  **Figure f4-jetem-5-3-i1:**
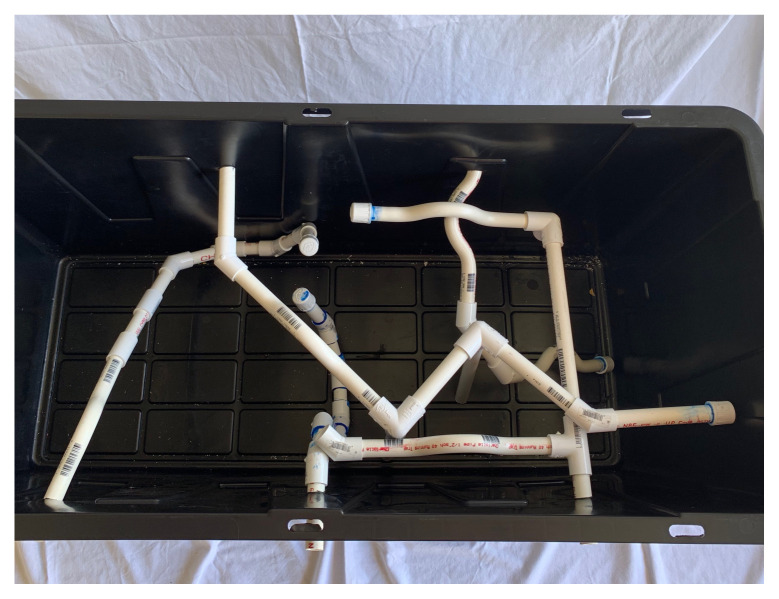

#### Simulated intubation ports

1. Using 1” Spade drill bit, drill two holes on tote lid.
2. Using ¾” PVC pipe, cut 2–3 ½” length and 2–5” pieces.
3. Using ½” PVC pipe, cut 2–12” pieces.
4. Place the 3 ½” x ¾” pieces through the drilled holes on the tote lid.
5. Using the 45 degree ¾” connector, glue the 3 ½” x ¾” piece to one end and the 5”x ¾” to the other end.
6. Take the 5” x ¾” end without the connector and glue on the ¾” PVC coupler.
7. At the ½” end of the connector, you may want to add some tape or cloth with a midline slit to represent vocal cords.
8. Place the ¾” side of the ¾” x ½” PVC connector into the open end of the coupler just glued into the apparatus.
9. Take the 12” ½” PVC piece and glue it into the ½” side of the ¾” x ½” PVC connector.
10. Glue on the ½” cap at the end of the apparatus.[Fig f5-jetem-5-3-i1]
  **Figure f5-jetem-5-3-i1:**
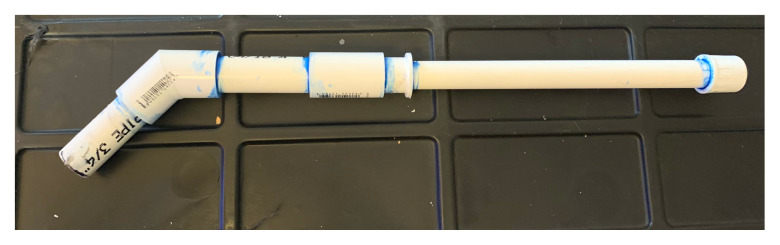
11. Repeat steps 1–10 in order to build the second simulated intubation apparatus.

### Associated content

Fiberoptic Intubation Checklist

### Results and tips for successful implementation

Residents were asked to take a survey before and after the airway simulator training. There were 4 questions on the survey. Number of fiberoptic intubations performed outside of simulation, confidence in being able to identify airway landmarks, confidence in being able to perform the procedure independently, and confidence in being able to identify the correct supplies necessary to perform a fiberoptic intubation. Confidence on the procedure was measured on a scale of 1–10, with 10 representing a feeling of complete confidence in performing the procedure on their own and 1 having no knowledge of the procedure. Residents included years PGY I–III who were present during the residents’ weekly academic conference.

8 PGY-I’s, 7 PGY-II’s, and 8 PGY-III’s completed the pre-simulation survey; 7 PGY-I’s, 5 PGY-II’s, and 6 PGY-III’s completed the post-simulation survey.[Fig f6-jetem-5-3-i1][Fig f7-jetem-5-3-i1][Fig f8-jetem-5-3-i1]

**Figure f6-jetem-5-3-i1:**
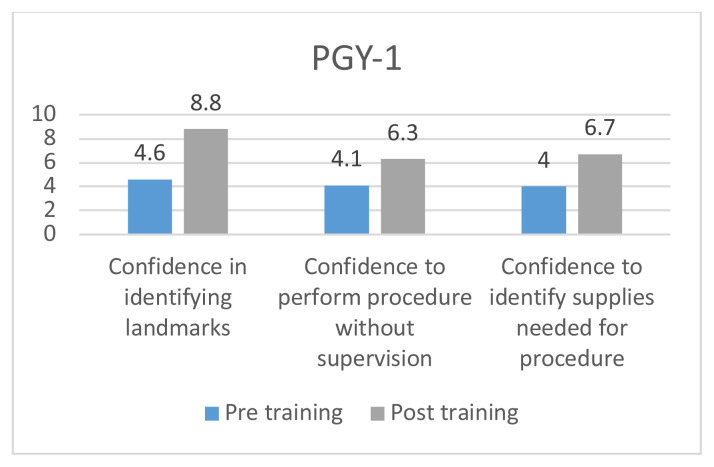


**Figure f7-jetem-5-3-i1:**
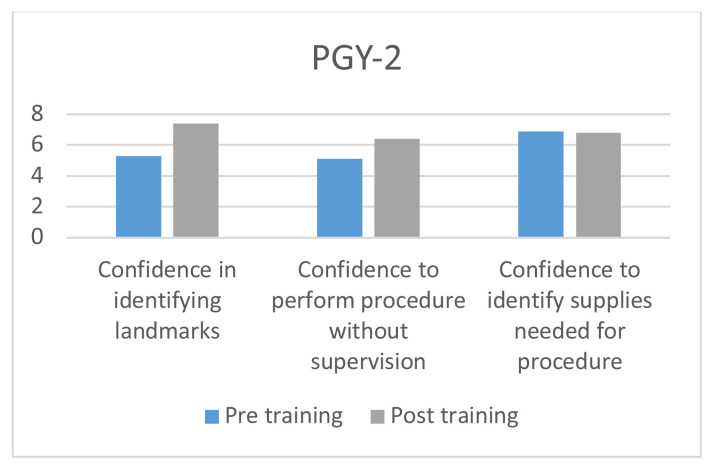


**Figure f8-jetem-5-3-i1:**
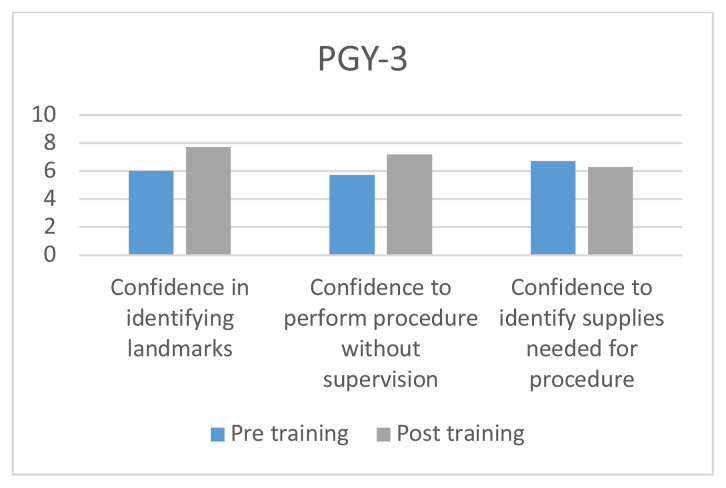


Overall there was a trend in increased confidence in performing the procedure without supervision, and identifying the landmarks across all three classes of residents. The greatest difference was seen among PGY I class with an improvement of 4.2 points in being able to identify the landmarks and a 2.2 increase in perceived ability to perform the procedure with confidence. There was a decrease in the confidence needed to identify the correct supplies needed for fiberoptic intubation among PGY II and PGY IIIs.

This trainer can be adapted in many ways. For example, in this specific learning session an additional maze was built in order to provide an opportunity to work through a portion with water, in order to simulate secretions or other liquid substances that could simulate a contaminated airway. This was a simple addition by gluing different PVC pipes together and drilling a small hole to add water in the maze. Many other options are available to make this trainer more specific for different sessions. In the future, options can be added to help with removing foreign bodies or building mazes to simulate different contaminated airways that would be seen in different cases.

### Discussion

It is possible that the act of reviewing and practicing hands on with the scope, and not necessarily the simulator itself that lead to the general increased confidence in being able to perform the procedure. Offering an interactive low-cost option to handle the equipment may encourage other learners to practice with this rarely used equipment. The benefit of our simulator is that it forces the user to manipulate the scope in ways more traditional trainers do not and increases familiarity with how the scope behaves.

There was not a large enough sample size to definitively demonstrate that this is an effective exercise, though there is a trend towards increased confidence, so further studies are needed. Collecting standardized surveys with objective checklists filled out by impartial observers across a range of training programs who choose to build the trainer may help determine if this is an effective exercise.

The decrease in ability to identify the necessary equipment, interestingly, was among the senior residents. On average, both the second- and third-year residents felt more confident in being able to identify necessary equipment, which may suggest they were overconfident in being able to identify the necessary equipment prior to the exercise. It is also possible this was a statistical error given the low sample size. The decrease may suggest that the simulation experience was too artificial, though one would expect decreases in confidence in being able to identify landmarks and perform the procedure unsupervised. The goal of the simulator was actually not to increase familiarity in being able to identify necessary equipment, and little of the exercise was dedicated towards this. More time was spent having residents use the scopes and the maze.

## Figures and Tables

**Table t2-jetem-5-3-i1:** 

Details of procedure	Yes	No
Mentions indications, complications, and contraindications for the procedure.		
Discusses the different approaches to fiberoptic intubation.		
In an awake intubation discusses preparing upper airway in order to increase first attempt success.		
In nasal intubation discusses steps performed during procedure.		
Proper set up for procedure, including having all equipment at bedside.		
Preloads ETT on scope.		
Holds scope appropriately including keeping scope taut.		
Advances scope appropriately.		
Uses counterclockwise and clockwise rotations in order to maneuver scope through airway.		
Identifies epiglottis and vocal cords correctly.		
Passes scope through vocal cords with minimal trauma to vocal cords.		
Advances ETT properly over scope and successfully passes ETT through vocal cords.		
Removes scope while maintaining ETT in proper place.		
Discusses post procedure process including post procedure CXR and putting equipment in proper places for cleaning.		

## INSTRUCTOR GUIDE

### Fiberoptic Intubation Checklist

[Table t2-jetem-5-3-i1]

Comments and Feedback: ____________________________________________________________________

__________________________________________________________________________________________

**__________________________________________________________________________________________**

**__________________________________________________________________________________________**

__________________________________________________________________________________________

## References

[1] Hayden EM Pallin DJ Wilcox SR Emergency department adult fiberoptic intubations: incidence, indications, and implications for Training Acad Emerg Med 2018 25 11 1263 1267 10.1111/acem.13440 29701889

[2] Simma L Cincotta D Sabato S Long E Airway emergencies presenting to the paediatric emergency department requiring advanced management techniques Arch Dis Child 2017 102 9 809 812 10.1136/archdischild-2016-311945 28404553

[3] Stringer KR Bajenov S Yentis SM Training in airway management Anaesthesia 2002 57 10 967 83 10.1046/j.1365-2044.2002.02830.x 12358955

[4] Klatif R Bautista A Duan X Teaching basic fiberoptic intubation skills in a simulator: initial learning and skills decay J Anesth 2016 30 1 12 19 10.1007/s00540-015-2091-z 26493397

[5] Williams KA Harwood RJ Woodall NM Barker GL Training in fiberoptic intubation Anaesthesia 2000 55 1 99 100 10.1046/j.1365-2044.2000.01257.x 10594458

[6] Xinyuan D Dongfeng W Bautista A Assessment of reaching proficiency in procedural skills: fiberoptic airway simulator training in novices Open Access Medical Statistics 2011 1 45 50 10.2147/OAMS.S24625

[7] McGaghie W Issenberg S Cohen E Barsuk J Wayne D Does simulation-based medical education with deliberate practice yield better results than traditional clinical education? A meta-analytic comparative review of the evidence Academic Medicine 2011 86 6 706 711 10.1097/acm.0b013e318217e119 21512370 PMC3102783

[8] Østergaard ML Rue Nielsen K Albrecht-Beste E Kjær Ersbøll A Konge L Bachmann Nielsen M Simulator training improves ultrasound scanning performance on patients: a randomized controlled trial Eur Radiol 2019 29 6 3210 3218 10.1007/s00330-018-5923-z 30617476

[9] Seymour NE Gallagher AG Roman SA Virtual reality training improves operating room performance: results of a randomized, double-blinded study Ann Surg 2002 236 4 458 463 10.1097/00000658-200210000-00008 12368674 PMC1422600

[10] Blum MG Powers TW Sundarasan S Bronchoscopy simulator effectively prepares junior residents to competently perform basic clinical bronchoscopy Ann Thorac Surg 2004 78 287 291 10.1016/j.athoracsur.2003.11.058 15223446

[11] Boet S Bould MD Schaeffer R Learning fiberoptic intubation with a virtual computer program transfers to ‘hands on’ improvement Eur J Anaesthesiol 2010 27 1 31 35 10.1097/EJA.0b013e3283312725 19851113

[12] Ahlberg G Enochsson L Gallagher AG Proficiency-based virtual reality training significantly reduces the error rate for residents during their first 10 laparoscopic cholecystectomies Am J Surg 2007 193 797 804 10.1016/j.amjsurg.2006.06.050 17512301

[13] Andreatta PB Woodrum DT Birkmeyer JD Laparoscopic skills are improved with LapMentor training: results of a randomized, double-blinded study Ann Surg 2006 243 854 863 10.1097/01.sla.0000219641.79092.e5 16772789 PMC1570578

[14] Draycott T Sibanda T Owen L Does training in obstetric emergencies improve neonatal outcome? BJOG 2006 113 2 177 182 10.1111/j.1471-0528.2006.00800.x 16411995

[15] Driver BE Reardon RF Flexible Endoscopic Intubation Robert JR Custalow CB Thomsen TW Hedghes JR Roberts and Hedges’ Clinical Procedures in Emergency Medicine and Acute Care 7th ed Philadelphia, PA Elsevier 2019 93 95

